# Pharmacological counseling in hepatotoxicity induced by macitentan and selexipag:  a case report 

**DOI:** 10.1186/s13256-022-03571-9

**Published:** 2022-10-18

**Authors:** Mariangela Lattanzio, Marco Ferrari, Stefano Martini, Francesca Ceriani, Andrea Imporzani, Franca Marino, Roberto De Ponti, Marco Cosentino

**Affiliations:** 1 Pulmonary Hypertension Unit, Department of Heart and Vessels, Ospedale di Circolo and Fondazione Macchi, University of Insubria, Varese, Italy; 2 Center of Research in Medical Pharmacology, University of Insubria, Via Monte Generoso 71, 21100 Varese, Italy

**Keywords:** Pulmonary arterial hypertension, Liver injury, Oral endothelin receptor antagonists, Oral prostacyclin receptor agonists, Polymorphisms

## Abstract

**Background:**

Pulmonary arterial hypertension is a progressive, debilitating condition characterized by increased resistance in the pulmonary arterial circulation. Current treatments for pulmonary arterial hypertension include endothelin receptor antagonists such as bosentan, sitaxentan, ambrisentan, macitentan, and oral prostacyclin receptor agonists such as selexipag. Endothelin receptor antagonists have been associated with liver injury, while hepatotoxicity was not reported for selexipag. Although genetic variability has been indisputably associated with variability in drug response, no study has been designed until now to assess its effects on the pharmacokinetics of endothelin receptor antagonists or selexipag.

**Case presentation:**

We report the case of a 58-year-old female Caucasian patient with a dramatic increase in plasma levels of transaminases after treatment with macitentan and selexipag, drugs whose risk of causing liver injury has so far been considered limited. After therapy discontinuation, plasma levels of transaminases returned to baseline, thus suggesting a role of these drugs in the observed hepatotoxicity. After pharmacological counseling, we decided to introduce ambrisentan for the patient’s treatment. After 7 months of treatment, no liver injury has been reported. To evaluate the role of genetic factors in the observed hepatotoxicity, we genotyped the patient for single-nucleotide polymorphisms previously associated with macitentan, ambrisentan, or selexipag metabolism. We found a genetic profile associated with a poor metabolizer (PM) phenotype for *CYP2C8* and *CYP2C9*, key enzymes for elimination of both macitentan and selexipag. The reported results suggest that an allelic profile associated with low activity for *CYP2C8* and *CYP2C9* enzyme could be a potential risk factor for macitentan and selexipag-induced liver injury and could provide a possible marker for early identification of subjects at higher risk of developing hepatotoxicity.

**Conclusions:**

A multidisciplinary approach based on clinical evaluation, as well as pharmacological counseling and evaluation of the patient’s genetic profile, might be useful for identification of patients with a high chance of drug-induced liver injury, avoiding unnecessary risks in therapy selection and prescription.

## Introduction

Pulmonary arterial hypertension (PAH) is a progressive and debilitating condition characterized by increased resistance in the pulmonary arterial circulation. PAH may be caused by multiple etiological factors but through a common pathogenesis resulting in pulmonary vascular remodeling, with pulmonary arterial smooth muscle cell proliferation and hypertrophy, vasoconstriction, endothelial dysfunction, and *in situ* thrombosis [1]. Current treatments for PAH include several classes of drug, although no compelling evidence supports the superiority of one class over others. However, orally administered drugs, such as endothelin receptor antagonists (ERAs), including bosentan, sitaxentan, ambrisentan, macitentan, and phosphodiesterase-5 inhibitors (sildenafil, tadalafil) or GMP-cycle stimulators (riociguat), are often prescribed as first-line treatment in mild to moderate PAH [1, 2]. Prostacyclin receptor agonist selexipag and parenteral subcutaneous and inhaled prostanoids (epoprostenol, treprostinil, and iloprost) are administered in patients with severe disease or after failure of other therapies [3, 4].

Some ERAs have been associated with liver injury. Bosentan and sitaxentan, in particular, have been linked with clinically significant hepatocellular injury, including liver failure. In 2010, sitaxentan was withdrawn from the market due to concerns about its liver toxicity [5], and bosentan was found to cause elevation of both liver aminotransferases and bilirubin levels, in about 17% of patients [6]. Macitentan appears to be burdened by a lower risk of hepatotoxicity, with a single reported case of fulminant hepatitis [7]. Finally, ambrisentan has not been associated with hepatotoxicity [8]. Oral prostacyclin receptor agonist selexipag has not, so far, been associated with hepatic injury.

Most studies on the mechanisms of ERA-induced hepatotoxicity have focused on drug–drug interactions, reporting increased liver uptake or inhibition of cytochrome P450 enzyme activity. For this reason, the pharmacokinetics of ERAs is well known: intracellular intake of these drugs is mediated by the organic anion transporter polypeptide (OATP) family (1/B1/1B3) coded by *SLCO (1/B1/1B3)* genes, whereas elimination of ERAs involves both phase I and II metabolic enzymes. In particular, macitentan is metabolized by the cytochrome P450 system, mainly the *CYP2C* family (*CYP2C8* and *CYP2C9* isoforms) [9], whereas ambrisentan is mainly glucuronidated through UGT isoenzymes such as *UGT1A9* [10].

Moving onto the pharmacokinetics of selexipag, its drug elimination pathways partially overlap with those of macitentan, and selexipag is indeed oxidized to its inactive metabolites by *CYP2C8* [11].

Although a role of genetic variability has been associated with variability in drug response, few studies to date have evaluated the effects of single-nucleotide polymorphisms (SNPs) (i.e., DNA sequence variations occurring when a single nucleotide in the genome differs between paired chromosomes) on the metabolism of ERAs [12, 13], and none have focused on the role of genetic variability in the pharmacokinetics of macitentan and ambrisentan.

We report the case of a patient with a dramatic increase in plasma levels of transaminases after treatment with both macitentan and oral prostacyclin receptor agonist selexipag. After pharmacological counseling, ambrisentan was introduced, with no signs of hepatotoxicity. To evaluate the role of genetic factors in the observed hepatotoxicity, we studied the patient’s genetic profile for the main enzymes/transporters involved in macitentan, ambrisentan, and selexipag pharmacokinetics.

## Case presentation

A 58-year-old female Caucasian patient, complaining of increasing fatigue and severe dyspnea, was admitted to the emergency department of Ospedale di Circolo & Fondazione Macchi, in May 2020 for acute cardiac failure with peripheral edema, ascites, pleural, and pericardial effusion. Echocardiography findings were highly suggestive for precapillary pulmonary hypertension: severe right chambers dilation, D-shaped left ventricle, severe tricuspid regurgitation, high velocity of tricuspid regurgitation with pulmonary pressure estimated at 100 mmHg, dilated and not collapsing inferior vena cava, and pericardial effusion. Computed tomography angiography (CTA) of the chest excluded pulmonary embolism; high-resolution computed tomography (HRCT) ruled out parenchymal diseases and mediastinal lymphadenopathy; capillaroscopy documented a scleroderma pattern; antinuclear antibodies (ANA), anti-centromere antibodies (ACA), and anti-mitochondrial antibodies tested positive; HIV serology tested negative; hepatic echo-Doppler ruled out portopulmonary hypertension. Diffusing capacity of the lungs for carbon monoxide (DLCO) was not tested because of severe hypoxemia.

Right heart catheterization (RCAT) documented a mean pulmonary arterial pressure (PAP) of 58 mmHg, cardiac index (CI) of 1.9 L/min/m^2^, right atrial pressure (RAP) of 8 mmHg, pulmonary capillary pressure (PCP) of 8 mmHg, and pulmonary vascular resistance (PVR) of 16 Woods units (WU). A diagnosis of limited variant systemic sclerosis with severe PAH was made. Laboratory tests showed a monoclonal spike of IgG-λ. Aspartate aminotransferase (AST) (34 U/L), alanine aminotransferase (ALT) (34 U/L) and bilirubin levels (1 mg/dL) tested within normal ranges. *N*-terminal-pro hormone brain natriuretic peptide (NT-proBNP) tested positive for possible heart failure (4534 ng/L). Pulmonary veno-occlusive disease was ruled out as a cause of PAH by bronchoalveolar lavage (BAL). Continuous intravenous treatment with epoprostenol was proposed but refused by the patient, who was therefore treated with a high dose of furosemide before starting a specific oral therapy for PAH. At first, suspecting PVOD, sildenafil 20 mg BID was prescribed and titrated to 20 mg TID over 1 month, without reported adverse effects. After this, macitentan was prescribed at 10 mg OD. After 1 month, a significant increase in AST (485 U/L) and ALT (451 U/L) levels was found on routine analyses. Macitentan therapy was therefore suspended, with a gradual decrease in levels of transaminases. Liver ultrasound and complete viral serology showed no signs of specific diseases.

After suspending macitentan administration, the patient’s clinical condition worsened and sildenafil was replaced with tadalafil 20 mg OD, which was then titrated to 40 mg OD. In February 2021, with normalization of levels of transaminases, selexipag therapy was introduced with slow titration. After 2 months, at the dose of 800 mcg BID with selexipag, AST and ALT levels were increased (201 and 232 U/L). Selexipag therapy was suspended, and RCAT was performed, resulting in the following: mean PAP of 62 mmHg, CI of 2.6 L/min/m^2^, RAP of 6 mmHg, and PVR of 12.7 WU. The implant of a subcutaneous pump for treprostinil infusion was proposed but refused by the patient. In May 2021, with normalization of levels of transaminases and after a pharmacogenetics consultation, ambrisentan was prescribed (5 mg/die) in association with tadalafil (40 mg/die).

**Fig. 1:**
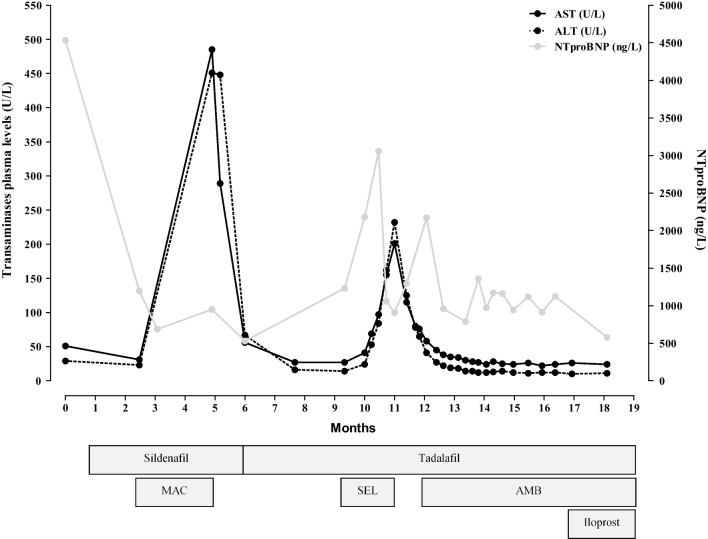
Flowchart of patient treatment. Evaluation of heart failure (by *N*-terminal-pro hormone brain natriuretic peptide measure) and liver injury (by transaminases plasma levels measure) risk related to pharmacological treatment. *MAC* macitentan, *SEL* selexipag, *AMB* ambrisentan, *AST* aspartate aminotransferase, *ALT* alanine aminotransferase, *NTproBNP* *N*-terminal-pro hormone brain natriuretic peptide

After the last RCAT (PAP 42 mmHg, CI 3.5 l/min/mq, RAP 6 mmHg, PVR 7.7 UW), the patient started triple therapy, adding inhaled iloprost. To date, good control of PHA symptoms has been achieved in the patient, without signs of liver injury (Fig. 1).

Genotyping of the patient (by real-time polymerase chain reaction using TaqMan probes) for SNPs associated with macitentan, ambrisentan, or selexipag metabolism found a genetic profile associated with a phenotype of poor metabolizer for both *CYP2C8* and *CYP2C9*, key enzymes for both macitentan and selexipag elimination, and intermediate metabolism phenotype for *UGT1A9*, the key enzyme for ambrisentan metabolism (Table 1).

**Table 1:** Patient genotype for selected SNPs

Gene	SNP	Nucleotide change	AF (%)	SNP effect	Patient genotype	Effects on metabolism/transport
*CYP2C8*	rs11572103 (*2)	c.805A>T	19	Decreased enzyme activity	*2/*2 (T/T)	↑↑
	rs11572080 (*3)	c.416G>A	12	Decreased enzyme activity	*1/*2 (G/A)	↑
	rs1058930 (*4)	c.792C>G	7	Decreased enzyme activity	*1/*4 (C/G)	↑
*CYP2C9*	rs1799853 (*2)	c.430C>T	10	Decreased enzyme activity	*2/*2 (T/T)	↑↑
	rs1057910 (*3)	c.1075A>C	5	Decreased enzyme activity	*1/*3 (A/C)	↑
*UGT1A9*	rs3832043 (*22)	(DT)9>10	40	Increased enzyme activity	*1/*1 (DT)9/9	=
	rs72551330 (*3)	c.98T>C	5	Decreased enzyme activity	*1/*1 (T/T)	=
*SLCO1B1*	rs2306283 (*1b)	c. 492A>G	50	Reduced transport activity	*1/*1 (A/A)	=
	rs4149056 (*5)	625T>C	14	Reduced transport activity	*1/*1 (T/T)	=

*AF* allelic frequencies in Caucasian populations

## Discussion

We report herein the occurrence of liver injury in a patient affected by PAH after treatment with macitentan and selexipag. So far, a single case of fulminant hepatitis has been reported in association with macitentan [7], while to the best of our knowledge, this is the first reported case of hepatotoxicity associated with selexipag. After pharmacological counseling, the patient was treated with ambrisentan with no signs of liver injury, thus confirming the hepatic safety of this drug [8].

To evaluate the role of genetic factors in the observed hepatotoxicity, we genotyped the patient for SNPs previously associated with low activity for enzymes important for macitentan, ambrisentan, or selexipag metabolism.

Metabolism of both macitentan and selexipag depends on the *CYP2C* family of enzymes, with partial overlap in their oxidation pathways [11, 14]. Ambrisentan, in contrast, follows a different metabolic pathway, which includes conjugation by *UGT1A9* [10].

In the *CYP2C9* gene, we found rs1799853 (*2) and rs1057910 (*3) SNPs, both of which are associated with a poor metabolizer (PM) phenotype [15] and with elevated levels of transaminases after treatment with ERAs (bosentan and sitaxentan) [14, 16, 17]. We hypothesize that the PM phenotype could lead to higher macitentan systemic exposure, thereby explaining the dramatic observed increase in plasma levels of transaminases. This result confirms the previously reported association between CYP2C9*2 variant and bosentan-induced liver injury [13].

Moving onto the *CYP2C8* gene, we found rs11572103 (*2), rs11572080 (*3), and rs1058930 (*4) SNPs, all of which are associated with a decrease in enzyme activity [18, 19], lower plasma levels for several drugs, such as paclitaxel, repaglinide, and ibuprofen, and lower arachidonic acid metabolism [18, 19]. *CYP2C8* contributes to macitentan elimination and is the key enzyme in selexipag metabolism [11]. An allelic profile associated with a PM phenotype for *CYP2C8* introduces, on the one hand, further evidence for the role of genetic factors in liver toxicity observed after macitentan treatment and, on the other hand, a possible explanation for hepatotoxicity observed also after treatment with selexipag. In this regard, selexipag has been shown to inhibit hepatic canalicular efflux transporters, such as breast cancer resistance protein (BCRP) [20], involved in bile acid excretion [11]. Therefore, the increase in selexipag availability and consequent BCRP inhibition can be hypothesized to cause intrahepatic bile acid accumulation and liver injury.

A key enzyme for ambrisentan metabolism is coded by the *UGT1A9* gene. Several SNPs have been identified for *UGT1A9*, among which two have functional consequences and quite high allelic frequency in Caucasian populations. rs3832043 (*1b), consisting in a (DT)10 insertion, determines higher enzyme expression compared with the (DT)9 allele [21], whereas rs72551330 (*3a) induces a large decrease in enzyme activity compared with wild type (named UGT1A9*1) [22]. A wild-type genotype for both SNP alleles was found in our patient. This allelic combination has been previously associated with an intermediate metabolism phenotype [23]. In view of this fact, the contribution of *UGT1A9* to ambrisentan elimination can be considered to be within normal range, thus explaining the observed good therapeutic response, with no increase in plasma levels of transaminases after ambrisentan treatment.

Functional variants in the *SLCO1B1* gene, encoding the *OATP1B1* transporter, were associated with liver uptake of ERAs and gallstone formation [24]. Our patient carried a wild-type genotype for both considered SNPs (rs2306283 and rs4149056), both associated with reduced transport activity. This is not surprisingly, since variants with reduced functionality would increase systemic exposure, more likely causing nonhepatic toxicities.

## Conclusion

We report a case of liver injury associated with both macitentan (a drug whose risk for hepatotoxicity is considered to be low [25]) and, for the first time, selexipag treatment. Moreover, we found that SNPs in *CYP2C8* and *CYP2C9* could be a potential risk factor for both macitentan- and selexipag-induced liver injury, thus providing a possible marker for early identification of subjects with high risk of developing hepatotoxicity. Obviously, dedicated clinical studies are required to validate this biomarker for the identification of PAH patients at higher risk for liver toxicity.

A multidisciplinary approach, based on not only clinical evaluation but also pharmacological and pharmacogenetics counseling, could be a first step toward “precision medicine” and might be useful to identify patients with increased risk of serious toxicity, avoiding unnecessary risks in therapy selection and prescription.

## Data Availability

Clinical data were retrieved from the patient’s medical files from the Department of Heart and Vessels, Ospedale di Circolo & Fondazione Macchi, University of Insubria, Varese, Italy. The genetic datasets used for the current study are available from the corresponding author on request.
